# Prospects of thermotolerant *Kluyveromyces marxianus* for high solids ethanol fermentation of lignocellulosic biomass

**DOI:** 10.1186/s13068-022-02232-9

**Published:** 2022-12-06

**Authors:** Priya Sengupta, Ramya Mohan, Ian Wheeldon, David Kisailus, Charles E. Wyman, Charles M. Cai

**Affiliations:** 1 Bourns College of Engineering, University of California Riverside (UCR), 900 University Avenue, Riverside, CA 92521 USA; 2 Center for Environmental Research and Technology (CE-CERT), Bourns College of Engineering, University of California Riverside (UCR), 1084 Columbia Avenue, Riverside, CA 92507 USA; 3 Department of Materials Science and Engineering, University of California at Irvine, Irvine, USA

**Keywords:** Biomass, Enzymes, Hydrolysis, Fermentation, High solids

## Abstract

Simultaneous saccharification and fermentation (SSF) is effective for minimizing sugar inhibition during high solids fermentation of biomass solids to ethanol. However, fungal enzymes used during SSF are optimal between 50 and 60 °C, whereas most fermentative yeast, such as *Saccharomyces cerevisiae*, do not tolerate temperatures above 37 °C. *Kluyveromyces marxianus* variant CBS 6556 is a thermotolerant eukaryote that thrives at 43 °C, thus potentially serving as a promising new host for SSF operation in biorefineries. Here, we attempt to leverage the thermotolerance of the strain to demonstrate the application of CBS 6556 in a high solids (up to 20 wt% insoluble solid loading) SSF configuration to understand its capabilities and limitations as compared to a proven SSF strain, *S. cerevisiae* D5A. For this study, we first pretreated hardwood poplar chips using Co-Solvent Enhanced Lignocellulosic Fractionation (CELF) to remove lignin and hemicellulose and to produce cellulose-enriched pretreated solids for SSF. Our results demonstrate that although CBS 6556 could not directly outperform D5A, it demonstrated similar tolerance to high gravity sugar solutions, superior growth rates at higher temperatures and higher early stage ethanol productivity. We discovered that CBS 6556’s membrane was particularly sensitive to higher ethanol concentrations causing it to suffer earlier fermentation arrest than D5A. Cross-examination of metabolite data between CBS 6556 and D5A and cell surface imaging suggests that the combined stresses of high ethanol concentrations and temperature to CBS 6556’s cell membrane was a primary factor limiting its ethanol productivity. Hence, we believe *K. marxianus* to be an excellent host for future genetic engineering efforts to improve membrane robustness especially at high temperatures in order to achieve higher ethanol productivity and titers, serving as a viable alternative to D5A.

**Supplementary Information:**

The online version contains supplementary material available at 10.1186/s13068-022-02232-9.

## Introduction

Plants store carbon in their secondary cell walls in the form of polysaccharides, viz*.,* cellulose, hemicellulose, and the aromatic polymer, lignin. These cell wall components can be converted via various biological and/or thermochemical routes into fuel ethanol, fuel additives, and/or specialty chemicals, or can be used as building blocks for synthesizing biopolymers [1]. Biological conversion of the sugars that make up the polysaccharides in plants via Simultaneous Saccharification and Fermentation (SSF) combines enzymatic hydrolysis of cellulose to glucose with fermentation of glucose to ethanol in a single step that promises the potential to realize nearly theoretical ethanol yields while taking advantage of powerful current and future biotechnological tools to facilitate its development [2]. Unlike Separate Hydrolysis and Fermentation (SHF), SSF reduces feedback inhibition caused by sugar accumulation, lowers the enzyme requirement, and avoids bacterial contamination, thereby facilitating economic ethanol production [3].

Despite recent progress in the development of producing high gravity sugar hydrolysates from biomass to support separate sugar fermentations, few studies have demonstrated high ethanol yields from SSF, particularly at solids loadings exceeding 10 wt%. However, high solids (glucan loading > 9 wt%) are needed in order to obtain ethanol titers over 50 g/L, a crucial yet elusive target to realizing significant reductions in energy and capital costs associated with ethanol recovery from the fermentation broth [4–6]. The increase in viscosity due to the high insoluble solid loadings required to reach these polysaccharide levels for substrates produced by many pretreatment systems results in inadequate mixing of the fermentation broth. This, in turn, leads to poor heat and mass transfer, while the build-up of sugars, ethanol, and lignin in the broth adversely impacts both enzyme activity and microorganism survival [7–9]. Moreover, biomass pretreatment, although largely dependent on type and severity of the technology used, can also produce a range of sugar and lignin degradation products, such as HMF, furfural, formic acid, acetic acid, and phenolics. Increasing the amount of pretreated substrate in the fermentation broth automatically increases the amount of these degradation products that can have a detrimental impact on the overall SSF performance [10–13].

Due to its recalcitrant nature, biomass can be first subjected to chemical or mechanical pretreatment in order to make its cellulose fraction more amenable to hydrolysis during SSF. Dilute acid-based pretreatments have been found to be highly effective in maximizing glucose yields while minimizing loadings of costly enzymes [14]. Here, we use Co-Solvent Enhanced Lignocellulosic Fractionation (CELF) pretreatment that applies co-solvent mixtures of THF and water in a 1:1 weight ratio with dilute (0.5 wt%) sulfuric acid at modest pretreament temperatures to achieve efficient lignin and hemicellulose removal while retaining a highly cellulose-enriched solid material for SSF operation (see materials and methods). The extensively delignified solids from CELF pretreatment have been found to be highly digestible by cellulolytic enzyme cocktails, demonstrating over 95% cellulose saccharification to glucose at enzyme dosages as low as 2 mg protein per g glucan in raw biomass [3, 15]. As we have demonstrated that nearly theoretical ethanol yields could be achieved by combining SSF with CELF pretreated biomass, resulting in final ethanol titers exceeding 85 g/L in one study [9, 15], this material would be particularly suitable to investigating the metabolic effects during high solids SSF.

Since commercial fungal-derived cellulolytic enzymes prefer a working range of 50–60 °C, while conventional yeast have an optimal growth range of 30–35 °C, SSF is typically conducted at an intermediate temperature of 37 °C to allow enzymes and yeast to both work effectively [16]. However, the reduced enzyme activity at the reduced temperature leads to a slower rate of sugar release than the rate of sugar consumption by the organism, eventually resulting in cell death by starvation [9, 17]. Thermotolerant organisms capable of fermenting sugars from a range of cellulosic substrates at temperatures close to 50 °C would offer two major benefits: 1) increased enzyme activity resulting in a faster rate of hydrolysis and fermentation and 2) reduced bacterial contamination due to the presence of ethanol, thereby saving additional costs for antibiotics [18–23].

*Saccharomyces cerevisiae* is one of the most extensively studied eukaryote, a widely used cell factory for numerous biotechnological applications, such as pharmaceuticals and proteins, and a valuable tool for research on eukaryotic organisms due to its easy acquiesce to genetic manipulation [24, 25]. It is also the most prominently used ethanologen for industrial ethanol production due to its high fermentative capacity, a high ethanol tolerance and excellent survivability in hyperosmotic conditions [26]. However, because growth of *S. cerevisiae* is limited to about 37 °C, its application in SSF requires use of lower temperatures than those preferred by fungal enzymes [27]. On the other hand, *Kluyveromyces marxianus* is a rather newly isolated non-model yeast strain procured from a range of habitats, including fermentated dairy products, sewage from sugar factories, and plants. Although, compared to *S. cerevisiae*, the accumulated knowledge of *K. marxinaus* is much smaller, however, because of its unique qualities of thermotolerance (up to 45 °C), high growth rate, the ability to grow on a broad spectrum of C5, C6 and C12 sugar substrates, and a high fermentative capacity, *K. marxianus* can potentially have a wide range of biotechnological applications, including cellulosic ethanol production [19, 28–37].

Pairing *K. marxianus* with CELF pretreatment could potentially unlock greater ethanol productivity at higher culture temperatures than what was possible with *S. cerevisiae*. In particular, the performances of D5A (a *S. cerevisiae* variant often used for SSF) and CBS 6556 (a *K. marxianus* variant that thrives at 43 °C) could be evaluated for SSF on real biomass without suffering negative substrate effects caused by biomass recalcitrance and mixing. Here, we demonstrate a unique study of application and comparison of the performance of CBS 6556 in high solids SSF configuration at an optimized temperature of 37 °C to a proven high performing S. cerevisiae D5A strain. We further subject CBS 6556 to a high solids SSF environment at a near saccharification temperature of 43 °C to truly leverage its thermotolerant capabilities in achieving high titers and yields of cellulosic ethanol at a much faster pace than the D5A strain.

## Results and discussion

### Growth, productivity, and sugar tolerance of *K. marxianus* and *S. cerevisiae* grown on glucose

**Fig. 1:**
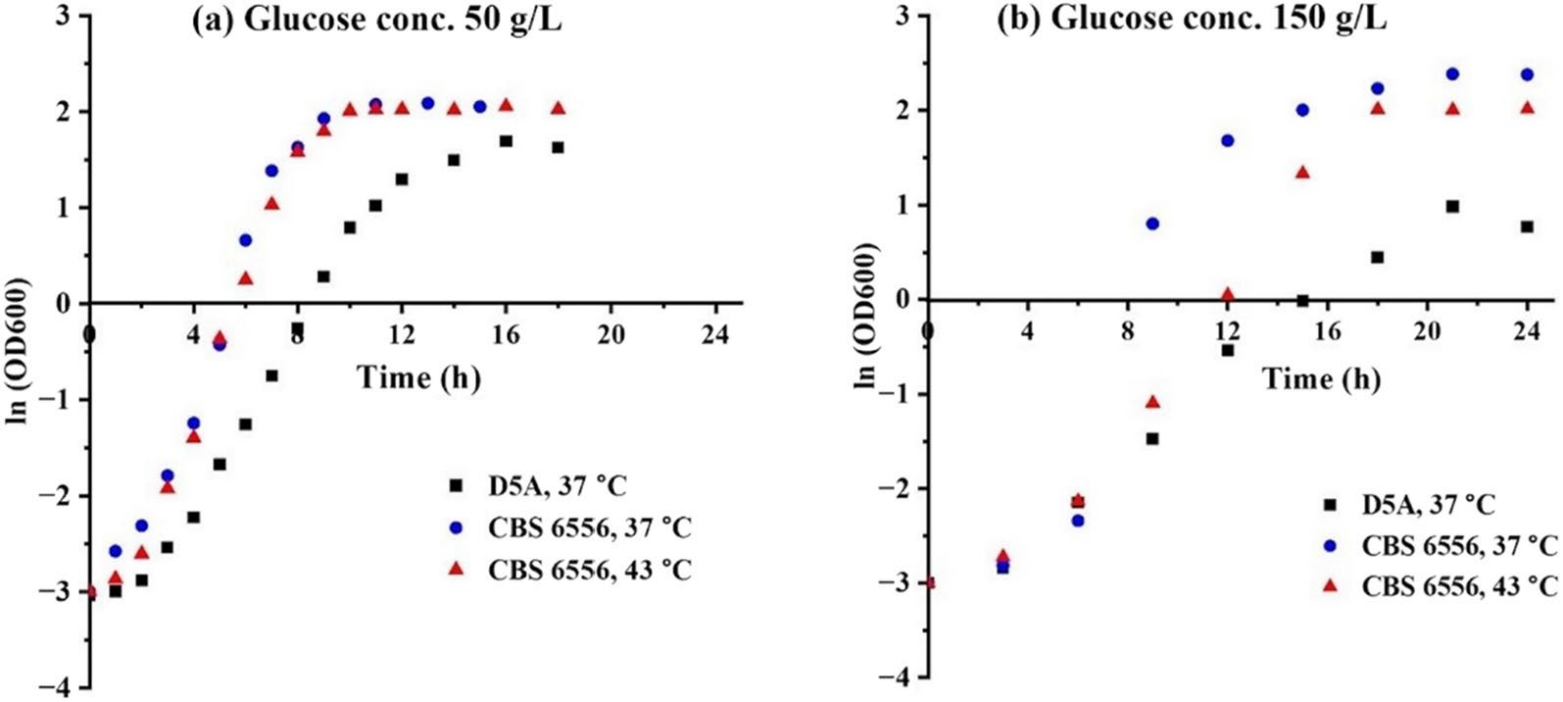
Anaerobic growth over time as measured by natural log of optical density measured at 600 nm wavelength (OD600) for the CBS 6556 strain of *Kluyveromyces marxianus* and D5A strain of *Saccharomyces cerevisiae* cultured in a shake flask on glucose concentrations of **a** 50 g/L and **b** 150 g/L with a 50 mL working volume at 37 and 43 °C

Sugars can quickly accumulate to very high concentrations during high solids SSF if the fermentative organism is unable to rapidly consume the sugars as they become hydrolyzed by enzymes. High sugar concentrations in the fermentation broth can, in turn, create hyperosmotic stress on the cells [26]. Coupling this stress with the need to operate at higher than optimal growth temperatures to foster sufficient enzyme action and ethanol accumulation results in osmotic, temperature, and ethanol stresses [38, 39]. To understand how these factors impact *K. marxianus* CBS 6556 and *S. cerevisiae* D5A, their growth and ethanol production were first evaluated by glucose fermentations when subjected to (i) a higher temperature, (ii) a high osmolarity, and (iii) evaluation of the combined effect of (i) and (ii). First, glucose concentrations of 50 and 150 g/L were fermented by both strains at 37 and 43 °C to determine how temperature and glucose concentration impacted performance. The results in Fig. 1 show that at 37 °C, CBS 6556 at a growth rate of 0.7938 and 0.6705 h^−1^ grew 1.7 times and 5 times faster than D5A at 0.4759 and 0.1318 h^−1^ for 50 and 150 g/L glucose concentrations, respectively. Thus, although both strains grew on both glucose concentrations, *K. marxianus* outperformed *S. cerevisiae* at 37 °C, a temperature typically employed to achieve reasonable enzyme activity in SSF. It is important to note that the growth of both CBS 6556 and D5A was hindered in the presence of high glucose at high temperature. However, the performance of D5A suffered much more under the combined stresses of temperature and higher glucose concentration. These data also reveal that *K. marxianus* maintained growth rates of 0.701 and 0.345 h^−1^at glucose concentrations of 50 and 150 g/L at 43 °C, while *S. cerevisiae* failed to grow at either concentration at this temperature. Another interesting observation from Fig. 1b is the reduced growth rate of CBS 6556 at 150 g/L glucose concentration at 43 °C which appears to be the result of a slow glucose metabolism exhibited by the strain at a higher temperature that prolonged the impact of hyperosmotic stresses, thereby impacting the growth. Overall, these results highlight the unique capabilities of CBS 6556 when compared to D5A and its potential to support higher temperature fermentation where fungal enzyme activity is also higher.

**Fig. 2:**
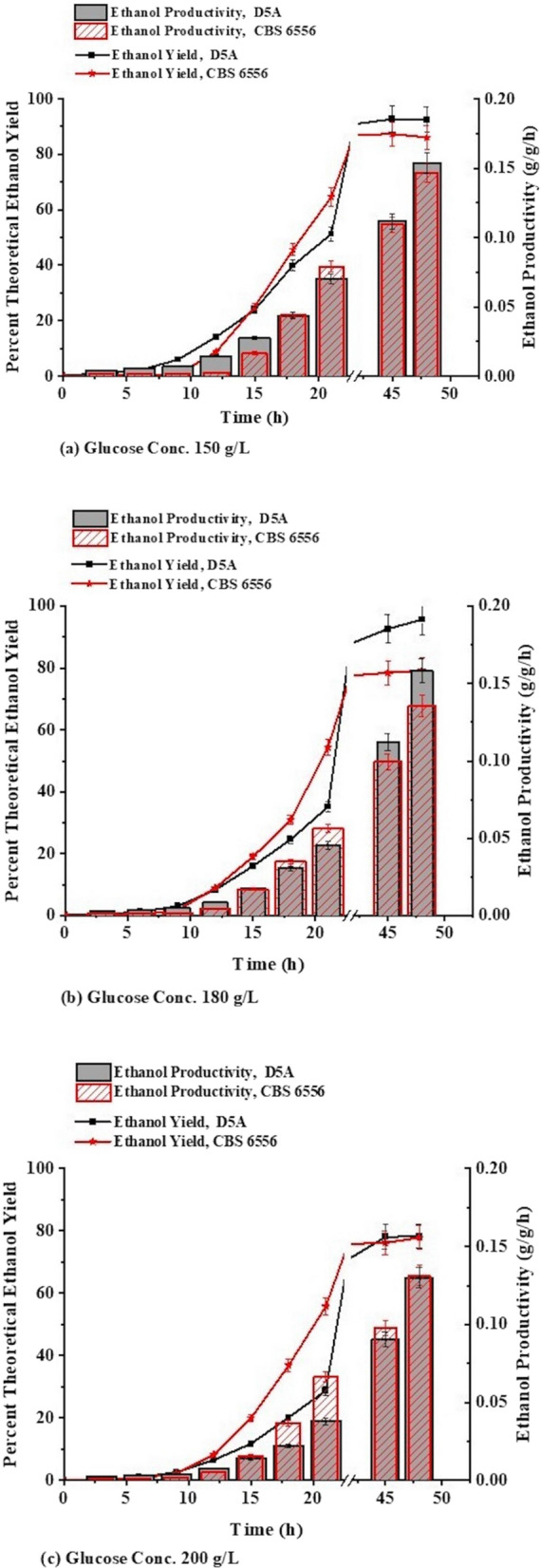
Percent of theoretical ethanol yields and ethanol productivities (g ethanol/g glucose fed/h) for growth of *K. marxianus* (CBS 6556) and *S. cerevisiae* (D5A) at 37 °C on glucose concentrations of (**a** 150, **b** 180, and **c** 200 g/L in a shake flask with a 50 mL working volume, in triplicates. Error bars indicated in the figure are standard deviation error bars among the triplicates

Next, the effect of glucose concentration on ethanol production by each organism was evaluated by fermenting glucose concentrations of 150, 180, and 200 g/L. As shown in Fig. 2, D5A and CBS 6556 both performed well for all glucose concentrations at 37 °C. These data also showed that CBS 6556 had a higher initial ethanol productivity at the larger glucose concentration, but performed similarly to D5A at other glucose concentrations. It is interesting to note that despite the slower growth rates for D5A shown in Fig. 1b, it was able to produce ethanol at a similar rate to the faster growing CBS 6556. Furthermore, after 2 days of glucose fermentation by both yeasts, concentrations of ethanol and glucose indicate that CBS 6556 left more glucose in solution than D5A for the two lower starting concentrations of glucose, while residual glucose reached almost 50 g/L for both strains when grown on 200 g/L glucose (Additional file 1: Table S1). The ethanol concentration from both yeasts did not increase significantly when the glucose concentration was raised from 180 to 200 g/L, as observed by the significant increase in residual glucose shown in Additional file 1: Table S1, suggesting that both yeasts were reaching an ethanol tolerance limit of about 80 g/L.

### Ethanol productivity and yields for high solids SSF of CELF pretreated poplar

In light of the glucose fermentation results, CBS 6556 and D5A would be expected to have similar ethanol tolerance and productivity and not be inhibited by the glucose concentrations expected in SSF. However, these results along with the higher growth rate, albeit on glucose, indicated that CBS 6556 should be more suitable than D5A for SSF at higher temperatures. To test whether these attributes would enhance SSF performance, each organism was employed for high solids SSF of CELF pretreated poplar. The CELF pretreated substrate in this study composed of 88.5% glucan, 3.0% xylan, and 2.3% acid-insoluble lignin. SSF experiments were conducted at 13, 17, and 20 wt% insoluble solids corresponded to 11, 15, and 18 wt% glucan-equivalent loadings. Both D5A and CBS 6556 were run at 37 °C, while CBS 6556 was also used in SSF at 43 °C to take advantage of the higher temperature tolerance displayed for glucose fermentations. A Cellic^®^ Ctec 2 enzyme cocktail was employed for each fermentation at a dosage of 15 mg protein per g glucan in raw poplar.

**Fig. 3:**
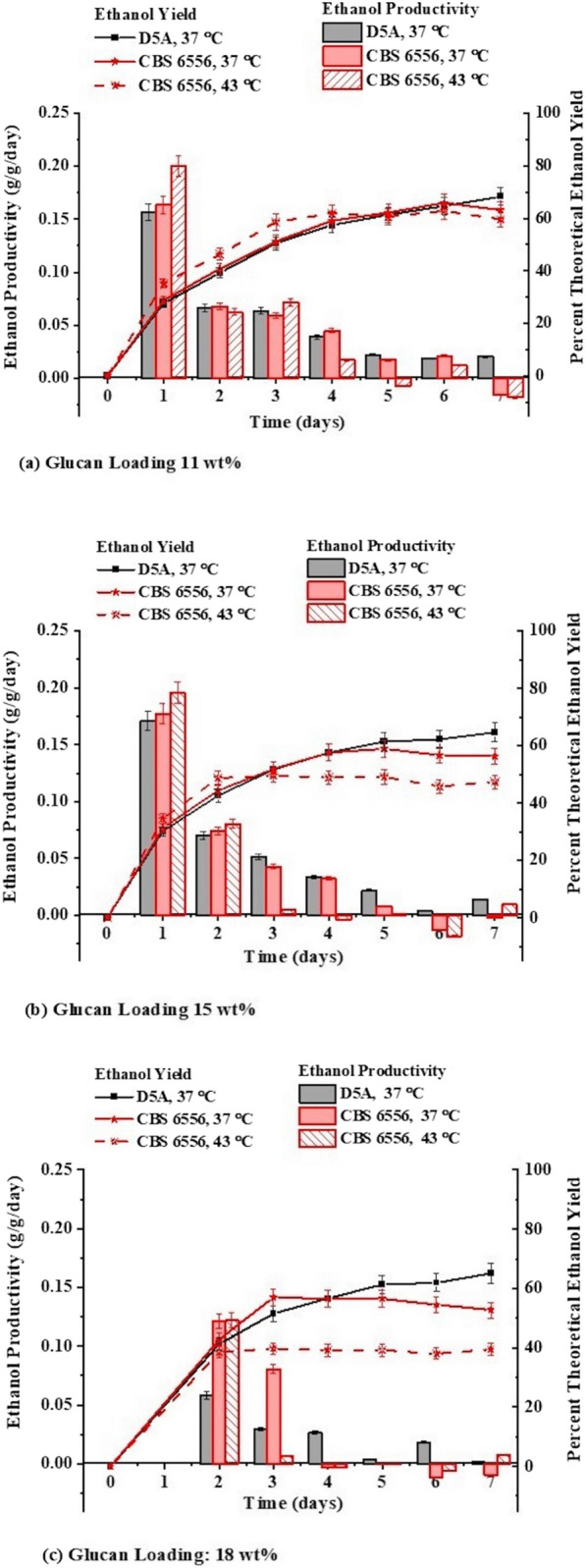
Ethanol productivities (g ethanol /g glucose equivalent of initial glucan/day) and percent of theoretical ethanol yields produced by *S. cerevisiae* (D5A) at 37 °C and *K. marxianus* (CBS 6556) at 37 and 43 °C when used for SSF of **a** 11, **b** 15, and **c** 18 wt% glucan loadings of poplar solids pretreated by CELF pretreatment. The SSF enzyme dose was 15 mg protein per g glucan for all cases. All the experiments were conducted in a shake flask with a 25 mL working volume, in duplicates. Error bars indicated in the figure are standard deviation error bars among the duplicates

Operation of CBS 6556 at 43 °C at 11 wt% glucan loading initially resulted in higher ethanol productivities (Fig. 3a), but 5-day yields for all three experiments were approximately the same (63%) and did not significantly increase at longer times. At a higher initial glucan concentration of 15%, the productivity of CBS 6556 at 43 °C was greater for an even shorter period of time (Fig. 3b) and when operated at 37 °C, both D5A and CBS 6556 had similar productivities up to day 5, after which D5A increased slightly while CBS 6556 leveled off. However, while the final yields for D5A at both 11 and 15 wt% glucan loadings were about the same, the yields dropped with increased glucan loadings for CBS 6556, particularly for operation at 43 °C. For application of SSF at 18 wt% glucan loadings, D5A demonstrated similar productivities and yields to those for both 11 and 15% glucan. On the other hand, although CBS 6556 operation at 37 °C closely followed the ethanol yields and productivities of D5A for the first 3 days, it virtually stopped ethanol production thereafter. The results show that the yield did not exceed 60% of the theoretical maximum and ethanol production ceased. Thus, these results show that operation of CBS 6556 at 43 °C exhibited the highest initial fermentation rates for 11 and 15 wt% glucan loading, potentially due to higher sugar release by cellulase operated nearer to its optimum temperature and the rapid ethanol production capacity of the strain. However, CBS 6556 also suffered from a much earlier fermentation arrest, likely due to the combined effects of higher ethanol concentrations and temperature.

**Fig. 4:**
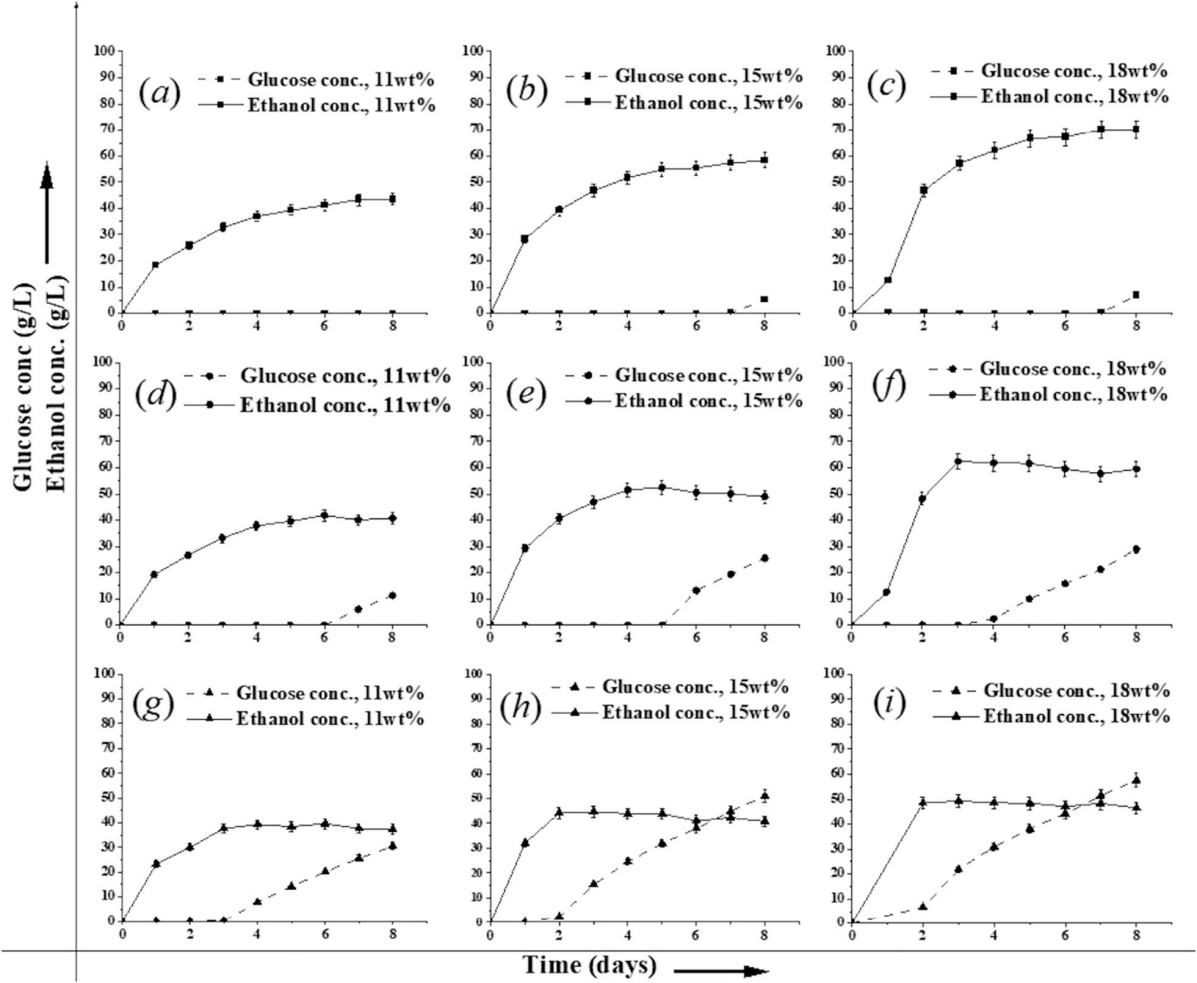
Ethanol and glucose concentrations (g/L) produced during SSF of CELF pretreated poplar solids by *S. cerevisiae* (D5A) at 37 °C (top layer) at glucan loadings of 11 (**a**), 15 (**b**), and 18 (**c**) wt% and *K. marxianus* (CBS 6556) at 37 °C (middle layer) at glucan loadings of 11 (**d**), 15 (**e**), and 18 (**f**) wt% and at glucan loadings of 11 (**g**), 15 (**h**), and 18 (**i**) wt% at 43 °C (bottom layer). All the experiments were conducted at an enzyme loading of 15 mg protein/per g glucan in raw poplar in a shake flask with a 25 mL working volume, in duplicates. Error bars indicated in the figure are standard deviation error bars among the duplicates

The results presented in Fig. 4 along with Additional file 1: Table S2 shed additional light on factors that caused a premature fermentation arrest during high solids SSF at 43 °C. As shown, D5A completely converted glucose released by the enzymes at 11 wt% glucan loadings and left only a little glucose in solution at the end of 15 and 18 wt% glucan run. On the other hand, when CBS 6556 was operated at the same temperature as D5A (37 °C), glucose accumulation started earlier and progressively increased with glucan loading to reach about 30 g/L at the two highest loadings. Furthermore, because ethanol production virtually stopped at the point glucose started building up, the greater amount of ethanol appeared to stop fermentation at these points. However, it is noteworthy that the final ethanol concentration increased with glucan loading, suggesting that faster glucose release from more glucan allowed 11 and 22% more ethanol to form at 15 and 18 wt%, respectively, as compared to 11 wt% before the fermentations stopped. Increasing the temperature to 43 °C resulted in glucose buildup much earlier in the fermentation and premature cessation of ethanol production at 4.3, 15.7, and 21% lower concentrations than achieved at 37 °C at increasing solid loadings. This points toward a reduced ethanol tolerance of CBS 6556 at 43 °C, since it can easily tolerate glucose concentrations > 50 g/L at 43 °C, Fig. 1b.

Overall, these results show that operation of CBS 6556 at 43 °C exhibited the highest initial fermentation rates for 11 and 15 wt% glucan, due to faster sugar release by cellulase operated nearer to its optimum temperature and simultaneous fermentation quickly carried out by the strain. However, CBS 6556 also suffered from a much earlier fermentation arrest due to the combined effects of higher ethanol concentrations and temperature. This outcome is consistent with results with *K. marxianus* strains capable of fermenting glucose and cane syrup at high temperatures of up to 47 °C that showed that although fermentation was rapid initially, the organism suffered from a rapid rate of cell death at higher temperatures in high gravity fermentations [18]. Other studies also observed a high temperature later-stage ethanol fermentation arrest by *K. marxianus* [40, 41].

### Impact of glucose, ethanol, and temperature on yeast

Yeasts, in general, are polymorphic organisms and can take many sizes and shapes, such as ellipsoidal, spherical, or elongated cylinders, depending on the environment to which they are exposed [42, 43]. Hyperosmotic stress, due to increased glucose concentration, results in rapid water diffusion from the yeast cells into the surrounding medium, thereby leading to loss of cell wall turgor pressure and cells shrinkage. Higher ethanol concentrations act adversely on the integrity of the cell membrane by increasing membrane fluidity and permeability that result in cellular ion leakage [44]. Ethanol also negatively impacts cell metabolism and inhibits cell growth and cell division [45]. In response to hyper osmolarity and ethanol shock, the cells can accumulate glycerol or other polyols, such as arabitol, mannitol, meso-erythritol, and/or xylitol to alter the equilibrium between the intracellular and extracellular environments and reduce diffusion of intracellular water [26, 39, 46, 47]. The result can be an increase in cell volume due to swelling [48, 49]. Heat shock, however, not only increases cell membrane fluidity but also causes protein damage, practically killing the organism unless it possesses heat shock proteins (HSPs), i.e., proteins that enhance thermotolerance of unicellular organisms, like yeasts and bacteria. HSPs usually protect thermally damaged proteins from accumulation, unfold aggregated proteins, and refold damaged proteins or efficiently degrade them [38, 50].

It is interesting to note that a certain amount of glycerol was coproduced along with ethanol by both D5A and CBS 6556 during the first 48 h of the high solids fermentation experiments that increased with the glucan loading, Additional file 1: Fig. S1. These results indicate toward an osmotic shock that was experienced by the cells in the beginning of the SSF possibly due to a faster saccharification rate as compared to the rate of glucose consumption/fermentation. Figure 4c and Additional file 1: Fig. S1-*c*, together show that while D5A produced some glycerol initially for SSF at 18% glucan-equivalent solids loadings, glycerol production was relatively unchanged as ethanol production continued at 37 °C due to no further glucose accumulation. At the same temperature, CBS 6556 coproduced glycerol along with ethanol, and glycerol production plateaued at a 50% higher level than for D5A when ethanol production ceased and glucose accumulation began, Fig. 4-f and Additional file 1: Fig. S1-*f*. Figure 4i and Additional file 1: Fig. S1-*i* also reveal that glycerol production similarly followed ethanol build-up for SSF by CBS 6556 at 43 °C and again leveled off when ethanol production stopped. However, the concentrations of ethanol and glycerol stopped building up at somewhat lower concentrations than for operation at 37 °C, despite a sufficient amount of glucose available in the broth to continue growth and fermentation. Additional file 1: Fig. S1 (*a* and *c*) reports that for SSF by D5A at 37 °C, glycerol concentrations increased by about 50% when glucan loadings were increased from 11 to 18 wt%. However, Additional file 1: Fig. S1 (*d* and *f*) shows that although glycerol levels reached a similar high value for SSF of 11 wt% glucan for CBS 6556 at 37 °C, the amount rose with increasing glucan loadings to reach about 250% of the amount at 18% glucan. Increasing the temperature to 43 °C for SSF by CBS 6556 resulted in a faster sugar release and a ~ 50% increase in the maximum glycerol produced with 11 wt% glucan loadings, Additional file 1: Fig. S1-*g*. At higher glucan loadings, despite the faster sugar accumulation, glycerol production increased but only modestly, Additional file 1: Fig. S1 (*h* and *i*). Overall, the lower glycerol concentrations produced by CBS 6556 at 18 wt% regardless of sugar accumulation suggests that it was unable to cope with the osmotic shock due to the added stresses of ethanol and high temperature than D5A.

**Fig. 5:**
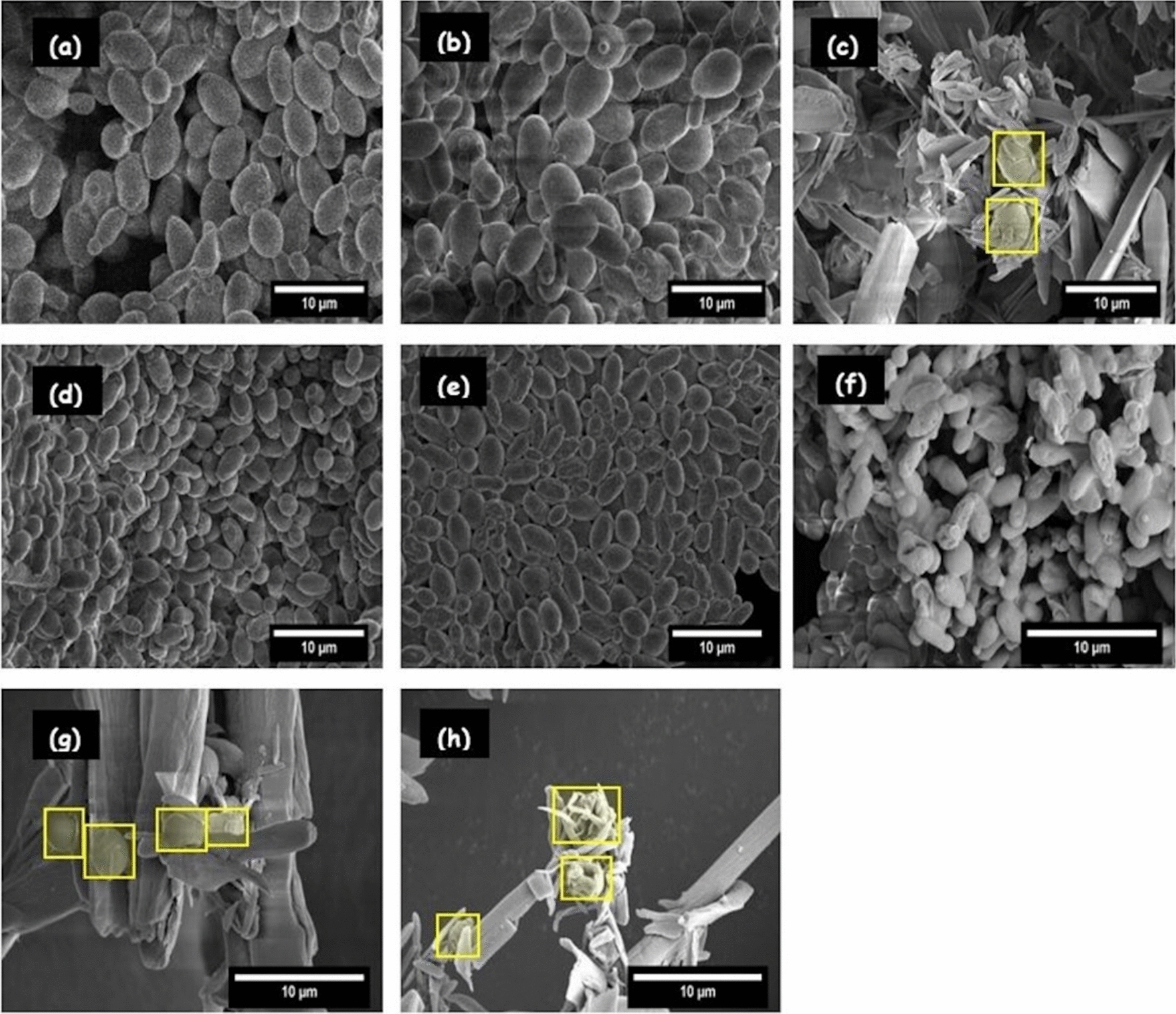
Scanning electron micrographs for anaerobic growth of *S. cerevisiae* D5A on **a** 50 g/L glucose at 37 °C, **b** 150 g/L glucose at 37 °C, **c** in SSF of CELF pretreated poplar with 18 wt% glucan loading at 37 °C, and of *K. marxianus* CBS 6556 on **d** 50 g/L glucose at 37 °C, **e** 150 g/L glucose at 37 °C, **f** 150 g/L glucose at 43 °C, **g** in SSF of CELF pretreated poplar at 18 wt% glucan loading at 37 °C and **h** SSF of CELF pretreated poplar at 18 wt% glucan loading at 43 °C (Magnification 10,000× at a voltage range of 2–5 kV.)

**Fig. 6:**
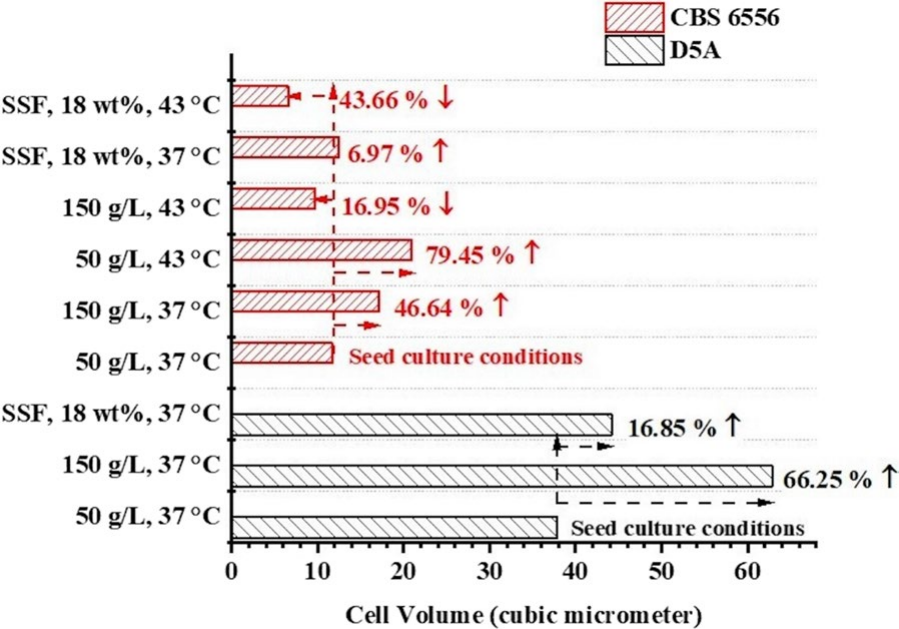
Calculated cell volume of *S. cerevisiae* D5A and *K. marxianus* CBS 6556 cells following pure glucose fermentations and SSF of CELF pretreated poplar, based on data collected from SEM images using ImageJ software

In order to further study the impact of temperature and ethanol concentration on CBS 6556 and D5A performance, electron micrographs were taken of both the strains at 48 h following fermentation of pure glucose and after 5 days following the SSF of CELF pretreated poplar. The conventional plate count method of estimating cell viability was avoided for these high solids fermentations with > 60% of the working volume comprising of wet fibrous biomass as it was logistically very difficult to isolate the cells from the biomass. As shown in Fig. 5a–h, both D5A and CBS 6556 cells maintained ellipsoidal or yeast-like morphologies when grown in an anaerobic environment. Therefore, we assumed the cells to be prolate ellipsoids and estimated their total surface areas and volumes based on their vertical and horizontal dimensions [12]. Although it was difficult to precisely image fibrous biomass and isolate the yeast cells in the SSF broth especially when the majority of the substrate has not undergone saccharification, it appeared that the oval structures highlighted in the yellow boxes (Fig. 5c, g, h) are similar in shape to the native ellipsoidal yeast. The cell volume estimations are calculated based on an elliptical geometry (Fig. 6). Figure 5f also reveals that CBS 6556 cells suffered substantial surface damage, including shrinking and wrinkling, likely due to greater shock at 43 °C compared to the behavior of this yeast (Fig. 5d) and D5A (Fig. 5b) under similar stresses at 37 °C. Figure 6 further indicates that when subjected to a 150 g/L glucose concentration at 37 °C, the cell volumes of D5A and CBS 6556 increased by 66.0% and 46.64%, respectively, as compared to their sizes at seed culture conditions. However, when subjected to similar ethanol concentrations at 43 °C, the average volume of CBS 6556 cells dramatically shrunk by almost 64.0%. These observations further indicate that CBS 6556 was more stressed by high concentrations of ethanol than D5A and the adverse impact was more pronounced at a higher temperature resulting in shrinking of CBS 6556 cells to an abnormally small size with quite noticeable surface damage.

Figure 6 shows similar observations from SSF of 18 wt% glucan at the end of 5-day glucose fermentations in that D5A and CBS 6556 volumes expanded by 16.8% and 6.97%, respectively, at 37 °C, while CBS 6556 contracted by 43.66% at 43 °C. However, as shown in Fig. 4f and Additional file 1: Table S2, the glucose concentration remaining at the end of 5 days of SSF at 43 °C was less than 50 g/L, a value within the tolerance limit of CBS 6556. This outcome indicated that the lower ethanol productivity could be attributed to reduced ethanol tolerance of CBS 6556 cells at higher temperatures [51].

Overall, these results suggest that CBS 6556 cells suffered major cell damage due to the combined effects of ethanol and heat shock. Because the cells were unable to make sufficient glycerol and/or maintain the turgor pressure of the cell wall, they shrunk to an abnormally small size. In addition, yeast cells need a critical size that is characteristic for the growth medium to initiate budding, and extremely small cells are incapable of budding, thereby arresting the cell cycle [22]. The atypically small cell size at high temperature and higher ethanol concentrations appeared to limit growth and metabolism of CBS 6556, thereby causing premature cessation of sugar uptake and fermentation at elevated temperature.

These observations are consistent with an analysis by Li et al. [41] of protein samples collected during *K. marxianus* fermentations at 45 °C that revealed some biochemical and enzymatic modifications triggered by stress conditions. They observed that some of the proteins related to gene transcription and translation, along with some of the proteins involved in oxidative phosphorylation, were down-regulated in *K. marxianus* after fermentation arrest. The repression of transcription and translation can be attributed to a self-defense mechanism to cope with stress condition during the late fermentation. Potentially, up-regulation of some molecular chaperones and proteasome proteins involved in the protein quality control (PQC) system after fermentation arrest could also be a limiting factor. The interactions of the proteins in the PQC system are responsible for the folding of proteins, refolding of misfolded proteins, and degradation of misfolded and damaged proteins. These observations provide some explanation for the observed fermentation halt and offer possible opportunities for metabolic engineering toward improvement of the stress tolerance in *K. marxianus*.

## Conclusion

Thermotolerant *K. marxianus* CBS 6556 was demonstrated in a high solids SSF configuration using CELF pretreated hardwood poplar to produce cellulosic ethanol. CBS 6556 was compared to *S. cerevisiae* D5A to demonstrate its potential for improved SSF performance at higher temperature fermentations. CBS 6556 achieved superior glucose consumption and ethanol productivity during early fermentation but did not achieve as high final ethanol titers and yields compared to D5A. CBS 6556 cells experienced an early fermentation arrest and underwent cell shrinkage, due to the combined stresses of elevated ethanol concentrations and temperature. Cross-examination of metabolite data between CBS 6556 and D5A and cell surface imaging revealed that loss of membrane integrity due to the combined stress of high temperature and high ethanol concentrations leads to the arrest of the cell’s metabolism. Overall, *K. marxianus* variant CBS 6556 showcased some extremely useful traits such as high growth rate along with a faster glucose consumption and ethanol production at both 37 and 43 °C that highlight its potential as a powerful future ethanologen. If the strain is engineered to tolerate > 10% (w/v) ethanol at 43 °C while maintaining the initial productivity of 0.125 g ethanol/g glucose equivalent of the initial glucan/day, it can reduce the effective fermentation time of SSF to half of that required by D5A to reach 90% ethanol yields, significantly reducing the operating cost of cellulosic ethanol production. However, the strain in its native form has some limitations to take maximum advantage of its thermotolerance to achieve favorable results. The observations presented in this study will help guide future genetic engineering efforts to improve ethanol tolerance in *K. marxianus* through membrane modification to allow it to sustain high ethanol productivity during SSF.

## Experimental section

### Materials

The woody biomass, *Populus trichocarpa*, also known as California Poplar, was generously provided by the BioEnergy Science Centre (BESC). The composition of the raw biomass as determined by following NREL LAP (version 08-03-2012) was 47.0% glucan, 16.9% xylan, and 21.2% acid-insoluble lignin [52]. The biomass was air-dried, knife milled using a laboratory mill (Model 4, Arthur H. Thomas Company, Philadelphia, PA, USA), and passed through a 1 mm internal sieve size. The enzyme cocktail used for the study was Cellic^®^ Ctec 2 generously provided by Novozymes^®^. Its protein content, as estimated using Pierce BCA analysis kit, was 250 mg/ml. The yeast strains used for fermentation were D5A, a variant of *Saccharomyces cerevisiae*, generously provided by the National Renewable Energy Laboratory (NREL), and CBS 6556, a *Kluyveromyces marxianus* strain obtained from the American Type Culture Collection (ATCC).

### CELF pretreatment

For CELF pretreatment of poplar wood chips, milled raw biomass was soaked overnight at 4 °C at a dry biomass loading of 7.5 wt% based on the total working mass in a 1:1 (weight basis) solution of THF: water, with 0.05 M H_2_SO_4_. The reactions were conducted in a 1 L Hastelloy Parr autoclave reactor (236HC Series, Parr Instruments Co., Moline, IL, USA) equipped with a double stacked pitch blade impeller rotating at 200 rpm. A series of CELF pretreatments were carried out at 160 °C for 15 min, i.e., conditions optimized for maximum total sugar recovery (not published). All reactions were maintained at temperature (± 2 °C) by convective heating using a 4 kW fluidized sand bath (Model SBL-2D, Techne, Princeton, NJ, USA), and the temperature inside the reactor was measured directly by an in-line thermocouple (Omega, K-type). At the end of the reaction, the reactor was cooled by submerging it quickly in a large water bath at room temperature. The solids were then separated from the reaction liquor by vacuum filtration at room temperature through glass fiber filter paper (Fisher Scientific, Pittsburgh, PA, USA). The mass and density of the liquid fractions were measured to calculate yields and close mass balances. The solids collected were then washed with (~ 150 mL) THF to remove residual lignin, followed by water washing until clear water ran through the solids. The solids were then hydraulically pressed to reduce the moisture content to 51.82%.

### Seed inoculum preparation

*K. marxianus* (CBS 6556) and *S. cerevisiae* (D5A) were both grown in 10 mg/mL yeast extract (Becton, Dickinson and Company, Redlands, CA), 20 mg/mL peptone (Becton, Dickinson and Company, Redlands, CA, USA), and 50 mg/mL glucose to the exponential phase and then stored in ~ 14 wt% glycerol. When needed for SSF, a frozen stock was thawed and grown overnight in 10 mg/mL yeast extract, 20 mg/mL peptone, and 50 mg/mL glucose in a 250 mL baffled flask shaking at 130 rpm in an incubator maintained at 37 °C. The inoculum was then centrifuged and re-suspended in sterile deionized (DI) water, and an inoculation was prepared at an optical density (O.D.) of 0.5 as determined at 600 nm.

### Pure sugar fermentations and growth curve

Pure sugar fermentations were carried out in 125 mL flasks at specified glucose concentrations. Glucose was dissolved in Millipore water and added to the flask and bubble trap assembly. Duplicates of those and a substrate blank were sterilized at 121 °C for 35 min in an autoclave and cooled in a laminar flow hood to prevent contamination followed by adding water to adjust for losses. 50 mM citrate buffer (pH 4.8) and 40 mg/L of tetracycline along with the seed inoculum were used in for 48 h fermentations shaking at 130 rpm and 37 °C for D5A and CBS 6556 and at 43 °C for CBS 6556. A 0.75 mL sample was taken every 2 h until stationary phase was reached, centrifuged at 15,000 rpm for 10 min, diluted, and analyzed to measure ethanol and sugar concentrations. Growth of the organisms was monitored by measuring the optical density at 600 nm wavelength of the fermentation broth sample at both aerobic and anaerobic conditions at required dilutions in a 1 mL cuvette with 1 cm path length using UV–Vis Spectrophotometer (SpectraMax ABS Plus, Molecular Devices, CA, USA). Growth rate, α (h^−1^), was measured by calculating the slope of the plot of ln (O.D.) versus time, *t*, using Eq. (1):

1 $$\ln \left( {\frac{{{\text{O}}.{\text{D}}{.}\,{\text{at }}\,{\text{time}} \left( {t + {\text{d}}t} \right)}}{{{\text{O}}.{\text{D}}{.}\,{\text{at}}\,{\text{time}}\,t}}} \right) = \alpha \left[ {( {t + {\text{d}}t)}) - t} \right].$$

### Simultaneous saccharification and fermentation (SSF)

Batch SSF experiments were performed in 125 mL flasks with a 25 mL total working volume containing CELF pretreated biomass corresponding to a desired glucan loading, 50 mM citrate buffer (pH 4.8), 40 mg/L tetracycline (Sigma Aldrich, St. Louis, MO, USA) as an antimicrobial agent, Cellic^®^ Ctec2 cocktail loaded at 15 mg protein per g glucan in raw poplar, and yeast inoculum. An assembly made with the flask and attached bubble trap was loaded with millipore water and the appropriate amount of substrate (Table 1). Duplicates with substrate along with a substrate blank assembly were sterilized at 121 °C for 35 min. The flasks were cooled in a laminar flow hood (Baker and Baker Ruskinn, Sanford, ME, USA) to prevent contamination, and reweighed to allow appropriate water replenishment. After adding the buffer, antimicrobial agent, enzyme cocktail, and yeast inoculum, SSF was carried out in flasks shaken at 130 rpm for 7 days at 37 °C for both D5A and CBS 6556 and at 43 °C only for CBS 6556. 1 mL samples were taken periodically, centrifuged at 15,000 rpm for 10 min, diluted, and analyzed to measure the sugar and metabolite concentration in the broth.

**Table 1:** Substrate loadings employed in SSF experiments

Case	Insoluble solid loading (wt%)	Corresponding glucan loading (wt%)	Enzyme dosage
1	13	11	15 mg protein per g raw glucan in raw poplar
2	17	15	
3	20	18	

### Measuring sugar and ethanol concentrations

Liquid samples along with appropriate calibration standards were analyzed by High-performance liquid chromatography **(**HPLC) (Waters Alliance 2695 system equipped with a Bio-Rad Aminex^®^ HPX-87H column and Waters 2414 RI detector) with a 5 mM sulfuric acid eluent flow rate of 0.6 ml min^−1^. The chromatograms were integrated using the Empower^®^ 2 software package (Waters Co., Milford, MA, USA).

### Model equations

At lower solid loadings, i.e., < 5 wt%, the density of the solvent phase was assumed to be the same as for just water. As the insoluble solid fraction increased, the density of the liquid fraction first increased due to increased sugar concentration and then slightly dropped due to the increasing ethanol concentration. Here, the modified version of the equations from Roche et al. [53] was employed to calculate the density of liquid fraction [54].

2 $${\text{Yield}}_{{{\text{Glucose}}}} = \frac{{C_{g} \times V_{l} /1.11}}{{M_{g} }} \times 100,$$

3 $$\% {\text{conversion}}_{{{\text{Glucan}}}} = \frac{{\left( {\frac{{C_{g} }}{1.11} + \frac{{C_{cb} }}{1.056} + \frac{{C_{Gly} }}{1.135} + \frac{{C_{Ac} }}{1.111} + \frac{{C_{Eth} }}{0.567}} \right) \times V_{l} }}{{M_{g} }} \times 100,$$

4 $$V_{l} = \frac{{M \times \left( {1 - S_{i} } \right)}}{{\rho_{l} }},$$

5 $$S_{i} = \frac{{S_{i0} - \left( {\frac{{\Delta C_{g} }}{1.11} + \frac{{\Delta C_{cb} }}{1.056} + \frac{{\Delta C_{x} }}{1.36} + \frac{{\Delta C_{Gly} }}{1.135} + \frac{{\Delta C_{Ac} }}{1.11} + \frac{{\Delta C_{Eth} }}{0.567}} \right)/\rho_{l} }}{{1 - \left( {\frac{{\Delta C_{g} }}{1.11} + \frac{{\Delta C_{cb} }}{1.056} + \frac{{\Delta C_{x} }}{1.36} + \frac{{\Delta C_{Gly} }}{1.135} + \frac{{\Delta C_{Ac} }}{1.11} + \frac{{\Delta C_{Eth} }}{0.567}} \right)/\rho_{l} }},$$

6 $$\rho_{l} = 0.456\left( {C_{g} + C_{cb} + C_{x} } \right) + 0.97,$$

7 $${\text{Theoretical}}\,{\text{ Ethanol}}\,{\text{ yield}} \left( \% \right) = {\raise0.7ex\hbox{${M_{Eth, g} }$} \!\mathord{\left/ {\vphantom {{M_{Eth, g} } {M_{g} }}}\right.\kern-\nulldelimiterspace} \!\lower0.7ex\hbox{${M_{g} }$}} \times 100{ } = {\raise0.7ex\hbox{${\left( {C_{Eth} \times V_{l1} \times 0.9} \right)}$} \!\mathord{\left/ {\vphantom {{\left( {C_{Eth} \times V_{l1} \times 0.9} \right)} {\left( {0.51 \times M_{g} } \right)}}}\right.\kern-\nulldelimiterspace} \!\lower0.7ex\hbox{${\left( {0.51 \times M_{g} } \right)}$}},$$

where $$C_{g} = {\text{Glucose Concentration, g/mL,}}$$, $$C_{cb} = {\text{Cellobiose Concentration, g/mL}}$$, $$C_{x} = {\text{Xylose Concentration, g/mL}}$$, $$C_{g} = {\text{Glucose Concentration, g/mL}}$$, $$C_{Gly} = {\text{Glycerol Concentration, g/mL}}$$, $$C_{Ac} = {\text{Acetic Acid Concentration, g/mL}}$$, $$C_{Eth} = {\text{Ethanol Concentration, g/mL}}$$, $$M = {\text{Initial mass of the system }}\left( {\text{Solids + Liquids}} \right)$$, g, $$M_{g} = {\text{Initial mass of glucan, g}}$$, $$V_{l} = {\text{Volume of the liquid phase, mL}}$$, $$S_{i0} = {\text{Initial insoluble solid fraction}}$$, $$S_{i} = {\text{Insoluble solid fraction at time }}t$$, $$\rho_{l} = {\text{Density of liquid phase, g/cc}}$$, $$M_{Eth,G} = {\text{Mass of ethanol in glucan quivalents, g}}{.}$$

### SEM sample preparation

Approximately 2 mL of SSF broth was centrifuged at 2400 rpm for 5 min to concentrate yeast cells. The cells were then suspended in saline phosphate buffer to remove any residual media. Next the cells were fixed in 2.5% glutaraldehyde in 4-(2-hydroxyethyl)-1-piperazineethanesulfonic acid (HEPES) buffer for at least 48 h followed by a serial dehydration (i.e., exposure to a series of ethanol concentrations: 50, 75, 80, 85, 90, 95, 99, and 100% for 10 min at each step).

The dehydrated cells were then mounted onto SEM stubs with conductive carbon tape and air-dried. The cells were then sputter coated with Pt/Pd for 90 s using a Cressington 108 auto sputter coater.

### Scanning electron microscopy (SEM)

Samples were examined using scanning electron microscopy (NNS450 FEI; USA) under high vacuum over a voltage range of ~ 2 to 5 kV. Images were collected at 10,000× magnification.

### Image analysis

Cell diameters were measured by using the line tool and analyze/measure function of the Image J software package [55]. Length measurements were calibrated using the scale bars on the image and the scale function of the software. Yeast cells were assumed to be prolate ellipsoids, and their total surface areas and volumes were estimated using the following equations:

8 $${\text{Surface area of a prolate spheroid}} = 2\pi \left( {a^{2} + \frac{ab\alpha }{{\sin \left( \alpha \right)}}} \right),$$

in which a is the horizontal radius, b is the vertical radius, and $\alpha$ is the angular eccentricity calculated as

9 $$\alpha = \arccos \left( \frac{a}{b} \right),$$

10 $${\text{Volume of prolate ellopsoid}} = \frac{4}{3}\pi ab^{2} .$$

## Supplementary Information

**Additional file 1: Table S1.** Glucose and ethanol concentrations and corresponding percent of theoretical ethanol yields after 2 days of 37 °C fermentations of 150, 180, and 200 g/L glucose by *S. cerevisiae* D5A and *K. marxianus* CBS 6556. **Table S2.** Peak ethanol concentrations and yields along with the time of occurrence and glucose concentrations at the end of fermentation resulting from application of D5A at 37 °C and CBS 6556 at 37 and 43 °C to SSF of CELF pretreated solids at 13, 17, and 20 wt% solids loadings and corresponding glucan levels using Cellic® CTec2 enzyme at a loading of 15 mg protein/g glucan in raw poplar. **Figure S1.** Glycerol concentrations (g/L) produced during SSF of CELF pretreated poplar solids by *S. cerevisiae* (D5A) at 37 °C (top layer in blue) at glucan loadings of 11 (*a*), 15 (*b*), and 18 (*c*) wt% and *K. marxianus* (CBS 6556) at 37 °C (middle layer in green) at glucan loadings of 11 (*d*), 15 (*e*), and 18 (*f*) wt% and at glucan loadings of 11 (*g*), 15 (*h*), and 18 (*i*) wt% at 43 °C (bottom layer in red). All the experiments were conducted at an enzyme loading of 15 mg protein/ per g glucan in raw poplar in a shake flask with a 25 mL working volume, in duplicates. Error bars indicated in the figure are standard deviation error bars among the duplicates.

## Data Availability

All data generated or analyzed during this study are included in this published article (and its supplementary information files).
