# Supplementary material for: Prospects of thermotolerant Kluyveromyces marxianus for high solids ethanol fermentation of lignocellulosic biomass
Source: Biotechnol Biofuels Bioprod. 2022 Dec 6;15:134. doi: 10.1186/s13068-022-02232-9 (PMC9724321; doi:10.1186/s13068-022-02232-9)
Supplement: Supplementary file 1 — Additional file 1: Table S1. Glucose and ethanol concentrations and corresponding percent of theoretical ethanol yields after 2 days of 37 °C fermentations of 150, 180, and 200 g/L glucose by S. cerevisiae D5A and K. marxianus CBS 6556. Table S2. Peak ethanol concentrations and yields along with the time of occurrence and glucose concentrations at the end of fermentation resulting from application of D5A at 37 °C and CBS 6556 at 37 and 43 °C to SSF of CELF pretreated solids at 13, 17, and 20 wt% solids loadings and corresponding glucan levels using Cellic® CTec2 enzyme at a loading of 15 mg protein/g glucan in raw poplar. Figure S1. Glycerol concentrations (g/L) produced during SSF of CELF pretreated poplar solids by S. cerevisiae (D5A) at 37 °C (top layer in blue) at glucan loadings of 11 (a), 15 (b), and 18 (c) wt% and K. marxianus (CBS 6556) at 37 °C (middle layer in green) at glucan loadings of 11 (d), 15 (e), and 18 (f) wt% and at glucan loadings of 11 (g), 15 (h), and 18 (i) wt% at 43 °C (bottom layer in red). All the experiments were conducted at an enzyme loading of 15 mg protein/ per g glucan in raw poplar in a shake flask with a 25 mL working volume, in duplicates. Error bars indicated in the figure are standard deviation error bars among the duplicates. [file 13068_2022_2232_MOESM1_ESM.docx]

**Supplementary Information**

**Table S1.** Glucose and ethanol concentrations and corresponding percent of theoretical ethanol yields after 2 days of 37 °C fermentations of 150, 180, and 200 g/L glucose by S. cerevisiae D5A and K. marxianus CBS 6556.

| *Glucose*  *concentration (g/L)* | *Yeast*  *strain* | *Ethanol*  *concentration (g/L)* | *Percent of*  *theoretical*  *ethanol*  *yield* | *Glucose*  *remaining*  *(g/L)* |
| --- | --- | --- | --- | --- |
| *150* | *D5A* | *70.63* | *90* | *0.15* |
|  | *CBS 6556* | *66.23* | *86.08* | *13.63* |
| *180* | *D5A* | *84.39* | *90* | *0.15* |
|  | *CBS 6556* | *73.51* | *79.20* | *37.33* |
| *200* | *D5A* | *81.09* | *78.33* | *42.62* |
|  | *CBS 6556* | *79.98* | *77.91* | *48.35* |

**Table S2.** Peak ethanol concentrations and yields along with the time of occurrence and glucose concentrations at the end of fermentation resulting from application of D5A at 37 °C and CBS 6556 at 37 and 43 °C to SSF of CELF pretreated solids at 13, 17, and 20 wt% solids loadings and corresponding glucan levels using Cellic® CTec2 enzyme at a loading of 15 mg protein/g glucan in raw poplar.

| Insoluble solid loading (wt%) | Glucan loading (wt%) | Yeast | Temp.  (°C) | Peak Ethanol concentration (g/L) | Percent theoretical ethanol yield (%) | Fermentation time corresponding to the peak titer  (days) | Glucose remaining at the end of 8 days (g/L) |
| --- | --- | --- | --- | --- | --- | --- | --- |
| 13 | 11 | D5A | 37 | 43.00 | 69 | 7 | 0 |
|  |  | CBS 6556 | 37 | 41.80 | 66 | 6 | 11.27 |
|  |  |  | 43 | 40.00 | 62 | 3 | 30.62 |
| 17 | 15 | D5A | 37 | 58.21 | 66 | 8 | 5.26 |
|  |  | CBS 6556 | 37 | 53.00 | 59 | 5 | 25.47 |
|  |  |  | 43 | 44.70 | 50 | 2 | 51.00 |
| 20 | 18 | D5A | 37 | 70.00 | 65 | 7 | 6.96 |
|  |  | CBS 6556 | 37 | 62.00 | 57 | 3 | 28.93 |
|  |  |  | 43 | 49.00 | 40 | 2 | 57.57 |


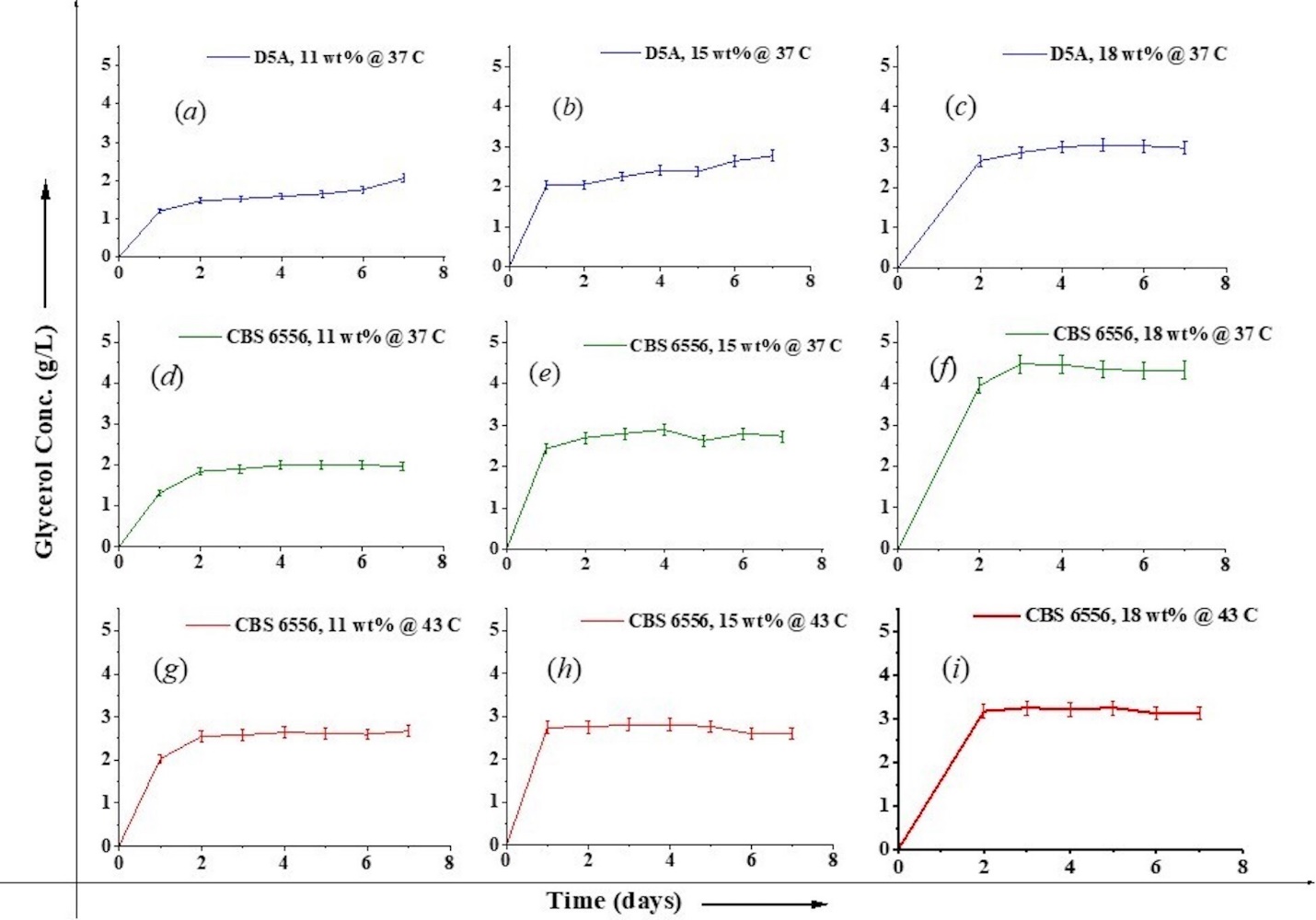


**Figure S1.** Glycerol concentrations (g/L) produced during SSF of CELF pretreated poplar solids by S. cerevisiae (D5A) at 37 °C (top layer in blue) at glucan loadings of 11 (a), 15 (b) , and 18 (c) wt% and K. marxianus (CBS 6556) at 37 °C (middle layer in green) at glucan loadings of 11 (d), 15 (e) , and 18 (f) wt% and at glucan loadings of 11 (g), 15 (h) , and 18 (i) wt% at 43 °C (bottom layer in red). All the experiments were conducted at an enzyme loading of 15 mg protein/ per g glucan in raw poplar in a shake flask with a 25 mL working volume, in duplicates. Error bars indicated in the figure are standard deviation error bars among the duplicates.
